# Indirect Determination of Mercury Ion by Inhibition of a Glucose Biosensor Based on ZnO Nanorods

**DOI:** 10.3390/s121115063

**Published:** 2012-11-06

**Authors:** Chan Oeurn Chey, Zafar Hussain Ibupoto, Kimleang Khun, Omer Nur, Magnus Willander

**Affiliations:** Physical Electronics and Nanotechnology Division, Department of Science and Technology, Campus Norrköping, Linköping University, SE-60174 Norrköping, Sweden; E-Mails: zafar.hussain.ibupoto@liu.se (Z.H.I.); kimleang.khun@liu.se (K.K.); omer.nour@liu.se (O.N.); magnus.willander@liu.se (M.W.)

**Keywords:** potentiometric inhibition biosensor, mercury, glucose oxidase, ZnO nanorods

## Abstract

A potentiometric glucose biosensor based on immobilization of glucose oxidase (GOD) on ZnO nanorods (ZnO-NRs) has been developed for the indirect determination of environmental mercury ions. The ZnO-NRs were grown on a gold coated glass substrate by using the low temperature aqueous chemical growth (ACG) approach. Glucose oxidase in conjunction with a chitosan membrane and a glutaraldehyde (GA) were immobilized on the surface of the ZnO-NRs using a simple physical adsorption method and then used as a potentiometric working electrode. The potential response of the biosensor between the working electrode and an Ag/AgCl reference electrode was measured in a 1mM phosphate buffer solution (PBS). The detection limit of the mercury ion sensor was found to be 0.5 nM. The experimental results provide two linear ranges of the inhibition from 0.5 × 10^−6^ mM to 0.5 × 10^−4^ mM, and from 0.5 × 10^−4^ mM to 20 mM of mercury ion for fixed 1 mM of glucose concentration in the solution. The linear range of the inhibition from 10^−3^ mM to 6 mM of mercury ion was also acquired for a fixed 10 mM of glucose concentration. The working electrode can be reactivated by more than 70% after inhibition by simply dipping the used electrode in a 10 mM PBS solution for 7 min. The electrodes retained their original enzyme activity by about 90% for more than three weeks. The response to mercury ions was highly sensitive, selective, stable, reproducible, and interference resistant, and exhibits a fast response time. The developed glucose biosensor has a great potential for detection of mercury with several advantages such as being inexpensive, requiring minimum hardware and being suitable for unskilled users.

## 1. Introduction

Among heavy metals, mercury metal, which is found in biological materials, natural water, soil, air, chemicals and waste products, is highly toxic, resulting in interference and disturbance of natural systems as well as the production of damaging effects to the environment [1]. The toxicity of mercury and its compounds produces harmful effects on the central nervous system and causes neuropsychiatric disorders [2], so the determination of mercury in environmental samples is therefore very important. There are several techniques which are normally used for the determination of mercury in environmental and biological samples, including atomic absorption spectrometry (AAS), stripping voltammetry, inductively coupled plasma spectrometry, cold vapour atomic fluorescence spectrometry (CVAFS), and cold vapour atomic absorption spectrometry (CVAAS), but these techniques require sample pretreatment, expensive instrumentation, complicated devices and require operation by skilled operators, hence these methods are not suitable for on-site testing and monitoring tasks [1–3]. Therefore, a simple new technology is needed with appropriate high capability to monitor mercury with fast response, while being inexpensive and making on-site monitoring possible. Biosensors are useful analytical tools for developing sensors in order to meet these requirements [4–6]. There are many types of biosensors for the detection of heavy metals such as purified protein-based, antibody-based, whole-cell-based, and both enzyme inhibition and activation-based methods [6]. Different electrochemical techniques are used for the detection of trace mercury in different enzyme reactions using different electrodes which have demonstrated great potential as effective tools to determine heavy metals in environment samples. Examples of these are the detection of mercury ion by an amperometric method using glucose oxidase immobilized on a carbon paste electrode [7], Pt electrode [8], thiolate self-assembled monolayer [9], poly-*o*-phenylenediamine (Pt/PPD/GOD) [10,11], cylindrical carbon film electrodes [12] and bienzyme electrodes based on their competitive activities [13]. Moreover, the determination of mercury ion using urease immobilized on the surface of an iridium oxide pehametric detector, self-assembled gold nanoparticles, nanostructured polyaniline-Nafion/Au/Al_2_O_3_ electrode and on polymeric membrane have been reported [14–17]. Furthermore, studies of mercury inhibition based on invertase enzyme immobilized on a copper-based electrode and GOD-modified platinum electrode [18] and a fast spectrometric method based on the inhibition of glucose-oxidase by mercury have been reported [19].

Table 1 lists some of the mercury determinations using different electrodes with immobilized glucose oxidase. From these reports, it is clearly observed that glucose oxidase is one of the most promising enzymes. It can be used for the indirect determination of mercury ions by different methods. However, there is no report using a ZnO nanomaterial in a study of mercury inhibition of glucose oxidase using a potentiometric technique. An advantage of the potentiometric technique over amperometric techniques is that for living biological samples, no current passes, and only the accumulation of charge is measured.

ZnO is a unique material that forms a diverse family of nanostructures such as nano-combs, nano-rings, nano-helixes, nano-bows, nano-belts, nanowires, and nano-cages and it exhibits multiple semiconducting, piezoelectric, and pyroelectric properties [20]. Furthermore, one dimensional (1D) ZnO nanostructures exhibit remarkable properties for sensing applications due to their high surface to volume ratio, high catalytic efficiency, non-toxicity, biocompatibility, chemical stability, strong adsorption ability because of the high isoelectric point (IEP ∼ 9.5) [21], bio-safety and high ionic characteristics (60%). In addition, ZnO does not dissolve at biological pH [22–24] and it also provides fast electron transfer properties [21,25]. Moreover, the advantages of using ZnO nanostructures for sensing applications are their high sensitivity, and time domain chemical sensing for low concentrations and the possibility of sensing in single cells or molecule detection available in small volumes at low concentration [26]. Such advantages cannot be achieved simultaneously using large sized sensors. Moreover, ZnO-NRs can be grown on flexible plastic substrates [27] which have excellent mechanical properties and can be suitable for medical and implantable biosensors. In this work, we have successfully presented the first potentiometric glucose biosensor made by the functionalization of a ZnO-NR array for studying the inhibition of mercury by glucose oxidase and perform simple and rapid determination of Hg^2+^ ions. The performance of the proposed sensor for both glucose detection and Hg^2+^ ion was monitored in test electrolyte solutions prepared in phosphate buffer solutions (PBS) having a pH of 7.4. Finally, the detection of other metals ions via a process of inhibition of the enzyme activity has been evaluated. In addition to this, the reproducibility of the enzymatic activity of the sensor was also examined for a glucose biosensor application.

## 2. Material and Methods

### 2.1. Reagents

Glucose oxidase from *Aspergillus niger* with activity of 280 units/mg, chitosan (C3646), D-(+)-glucose (99.5%), zinc nitrate hexahydrate, mercury(II) chloride and hexamethylenetetramine (HMT), acetic acid, were purchased from Sigma-Aldrich (Stockholm, Sweden). Phosphate buffer solution (PBS, 10 mM) was prepared by mixing 8 mM of Na_2_HPO_4_, 1.5 mM of KH_2_PO_4_, 0.135 mM of NaCl and 2.7 mM of KCl in deionized water and then the pH was adjusted to 7.4. A stock solution of 100 mM glucose was prepared in PBS and stored at 4 °C and 100 mM of mercury(II) chloride was prepared in deionized water. The low concentration standard solutions of both glucose and the mercury were freshly prepared before the measurements.

### 2.2. Fabrication of Glucose Biosensor Electrodes

The ZnO-NR array electrodes were prepared on a glass substrate by first evaporating titanium (Ti) as an adhesion layer and then followed by gold (Au) films with a thickness of 10 nm and 100 nm, respectively using an evaporation system (Evaporator Satis CR725). Then these gold coated glass electrodes were ultrasonically cleaned with isopropanol followed by rinsing in deionized water and then dried in air at room temperature. Then, ZnO-NRs were grown on the electrode by using the low temperature ACG approach as described in [28,29]. In this paper, the gold coated glass electrodes were spin coated in two steps with a seed solution containing zinc acetate using a speed of 1,000 rpm for 10 s and 2,500 rpm for 20 s, respectively, and then annealed at 120 °C for 10 min. After that the electrodes were placed horizontally in an aqueous solution of 0.025 M zinc nitrate hexahydrate [(Zn(NO)_3_)_2_·6H_2_O)] and 0.025 M hexamethylenetetramine [C_6_H_12_N_4_] and kept in a preheated oven for 5 h at 80 °C. When the growth was completed, the grown ZnO-NRs were cleaned with deionized water and dried at room temperature. It should noticed that at the top of the gold coated glass substrates were partially covered and the covered part was used as contact area.

### 2.3. Enzyme Immobilization on ZnO-NRs Arrays

The GOD was immobilized on the ZnO-NRs using a mixed solution consisting of 1 mL of GOD solution prepared by dissolving 10 mg/mL of GOD in PBS with a pH of 7.4 and 1 mL of chitosan membrane in order to improve the stability and activity of the enzyme on the electrode surface. The chitosan is used as the matrix for the immobilization of the enzyme due to its good properties, which include an excellent membrane-forming ability, high permeability toward water, good adhesion, biocompatibility, non-toxicity and high mechanical strength [30]. The chitosan membranes were prepared as described in [31]. Next 0.1 mL of a glutaraldehyde (GA) solution (2.5%) was added into the GOD-chitosan solution. Then the electrostatic physical adsorption method was applied for the immobilization of GOD on the ZnO-NR electrode due to the fact the chemical structures of GOD and ZnO both have polar atoms which can easily be attracted through electrostatic binding. The enzyme was electrostatically immobilized on the electrodes by dipping the electrodes into the mixture of the above solution for 5 min, then it was dried in air at room temperature for 1 hour. The immobilized electrodes were kept in dry conditions at 4 °C when not in use.

### 2.4. Electrochemical Measurements of the Proposed Mercury Ion Sensor

All the measurements of the glucose oxidase-based mercury ion sensor, with and without inhibitor, were performed at room temperature by using a pH meter (Model 744, Metrohm). The used Ag/AgCl reference electrode was purchased from Metrohm (3 M KCl). The response time was measured by a Keithly model 2400 series, which can provide the sensed electrochemical output *vs.* time.

## 3. Results and Discussion

### 3.1. Morphology and Chemical Composition of the Electrode

**Figure 1.**
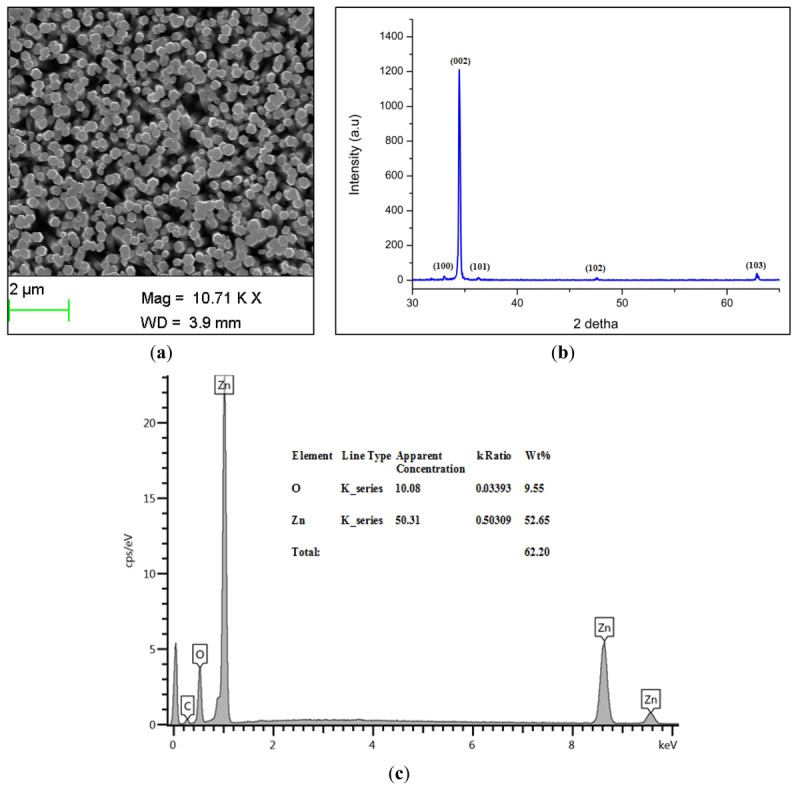

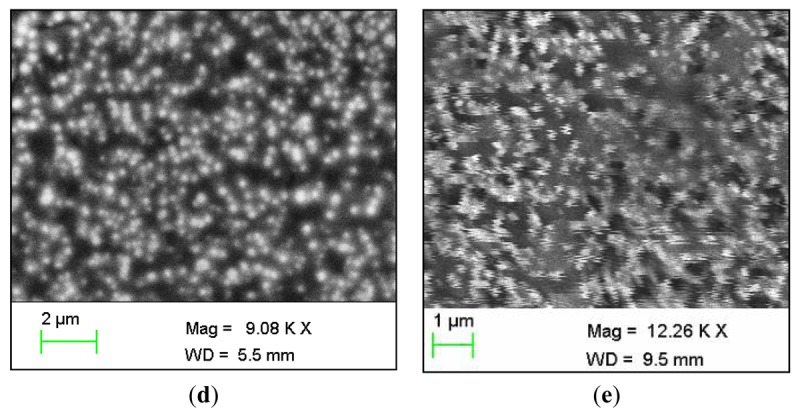
(**a**) SEM image of ZnO-NRs (**b**) XRD spectra of ZnO-NRs (**c**) EDS spectra of ZnO-NRs, (**d**) SEM image of the immobilized GOD on ZnO-NRs, (**e**) SEM image of the used electrode.

Scanning electron microscope (SEM) images of the grown ZnO-NRs are shown in Figure 1(a), while the X-ray Diffraction (XRD) pattern of the grown ZnO-NRs is shown in Figure 1(b).

It is seen that the ZnO-NRs exhibit a hexagonal wurtzite crystalline structure. The observed peaks were 002, 100, 101, 102 and 103 planes. The highest intensity peak was the 002, demonstrating that the ZnO-NRs grow along the c-axis and perpendicular to the substrate [32]. The energy-dispersive X-ray spectroscopy (EDS) analyses for the chemical composition of grown ZnO-NRs is shown in Figure 1(c). It is clearly indicated that the grown ZnO-NRs are composed of Zn and O atoms only and no other impurity was found. The immobilized ZnO-NRs arrays are shown in Figure 1(d). After the immobilization of enzyme it is seen that it completely covers the ZnO-NRs providing an extra protection for the ZnO-NRs from dissolving. Moreover, this figure gives evidence for the presence of glucose oxidase on the whole surface of ZnO-NRs. Finally, the SEM image of the used electrode is shown in Figure 1(e). It was revealed that the enzyme is still covered on the electrode and the ZnO nanorods were not dissolved after the electrodes were used.

### 3.2. Interaction between Chitosan and Glucose Oxidase

**Figure 2.**
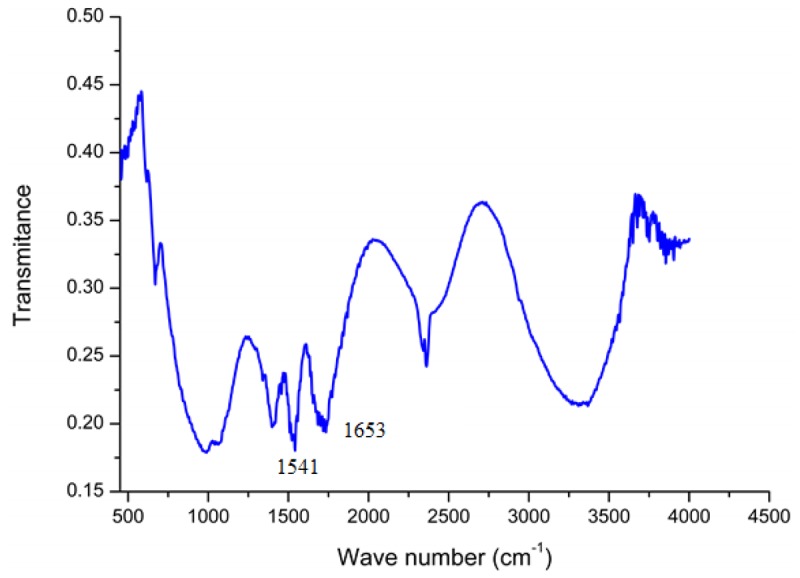
Fourier transform infrared (FTIR) spectrum of glucose oxidase immobilized on the ZnO-NRs.

In order to confirm the presence of GOD on the surface of ZnO-NRs, a Fourier transform infrared (FTIR) study was carried out. The most important observed IR peaks for the GOD are the amide I band (around 1,650 cm^−1^) and amide II band (around 1,540 cm^−1^) of the amide group and this spectrum is almost identical to the IR spectrum of chitosan, which shows these peaks at 1,654 cm^−1^ and 1,597 cm^−1^ [33]. However two weak peaks also appeared below 1,400 cm^−1^ which can assigned to the carboxylate groups in the enzyme. The measured FTIR spectrum of GOD/ZnO-NRs shows infrared bands of amide I at 1,653 cm^−1^ (C=O) and amide II at 1,541 cm^−1^ (N–H and C–H) as shown in Figure 2. These interesting small changes from the natural spectrum of GOD indicated that the glucose oxidase has formed a good matrix with the chitosan membrane.

### 3.3. The pH Effect

**Figure 3.**
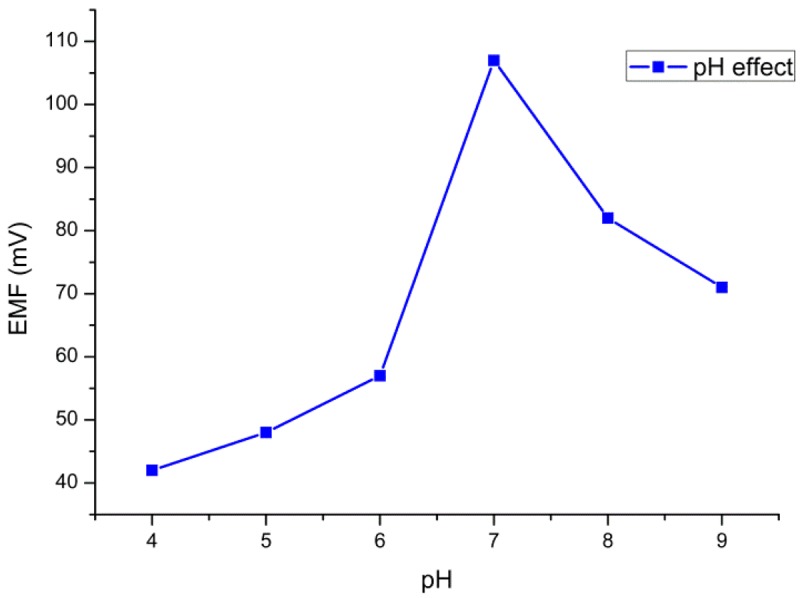
The pH effect on the potentiometric response of biosensor electrode.

The pH of the substrate solutions (glucose solution) can affect the overall enzymatic activity. Therefore the investigation of the effect of pH on the performance of the biosensor is very important. In this work, we recorded the potential response for 10 mM of glucose in PBS by varying the pH value from 4 to 9. The response of the sensor to the change of pH is shown in Figure 3. The highest activity of the enzyme was observed around pH 7. Therefore, pH 7.4 was adopted for all the measurements.

### 3.4. Temperature Effect

**Figure 4.**
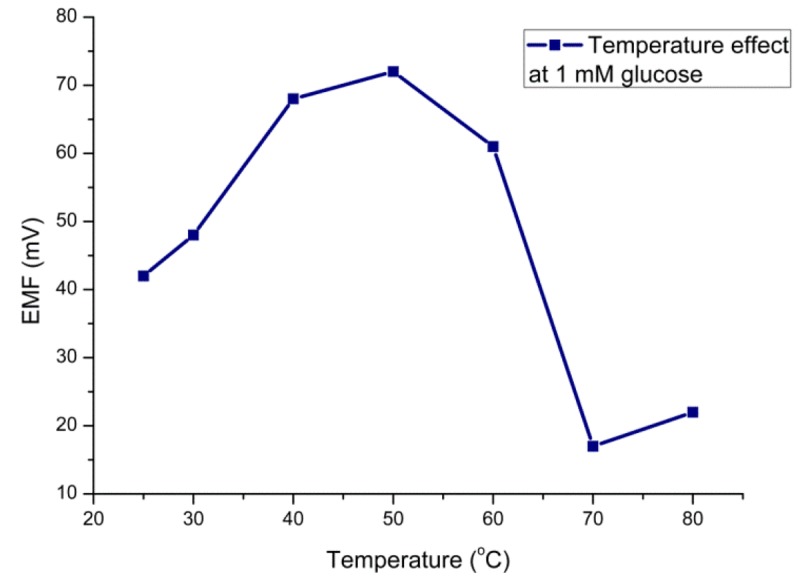
The temperature effect on the potentiometric response of biosensor electrode.

The efficiency of the glucose sensor with respect to the temperature can be explained in terms of the optimum temperature at which the sensor electrode would show the maximum performance. The sensor electrode has shown its maximum response around 50 °C (Figure 4) which can be attributed to the agitation of ions inside the testing solution with the increase in temperature. Although the optimum temperature for glucose oxidase is about 40 °C, we did all experiments at room temperature due to simplicity of the measurement and to avoid any possible evaporation processes. Moreover, the sensor electrode has shown a decreasing potentiometric response above 50 °C due to possible denaturation of the protein molecules at high temperature.

### 3.5. Measurement of Glucose Substrate

**Figure 5.**
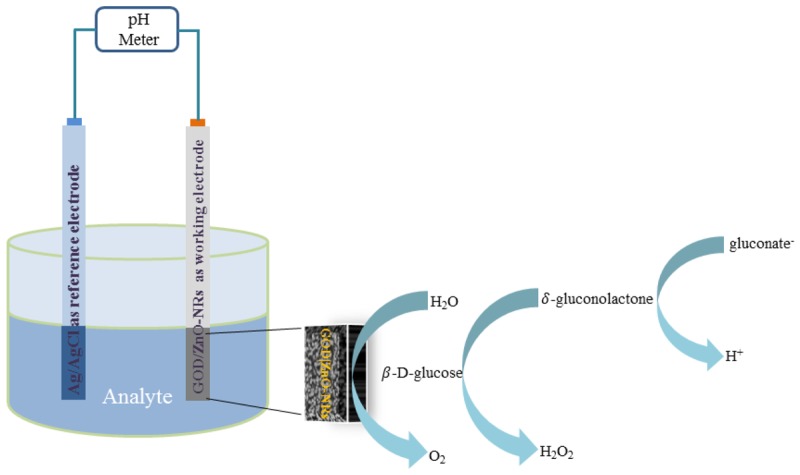
Schematic diagram of the sensing mechanism.

For the glucose sensing mechanism based on an enzymatic reaction catalysed by glucose oxidase (GOD) with β d–glucose gives the charged products gluconate^−^ and a proton (H^+^), according to Figure 5. The electromotive force (EMF) response of ion selective electrodes, the glucose biosensor, can be explained according to Nernst's expression:

(1) $$E+E^{0}+2.3\frac{\text{RT}}{\text{nF}}\log\left(\frac{\text{Ox}}{\text{Red}}\right)$$

where *E* is the cell potential at some moment in time, *E*° is the cell potential when the reaction is at standard-state conditions, *R* is the ideal gas constant in units of Joules per mole, *T* is the temperature in Kelvin, *n* is the number of moles of electrons transferred in the balanced equation for the reaction, and *F* is the charge on a mole of electrons.

**Figure 6.**
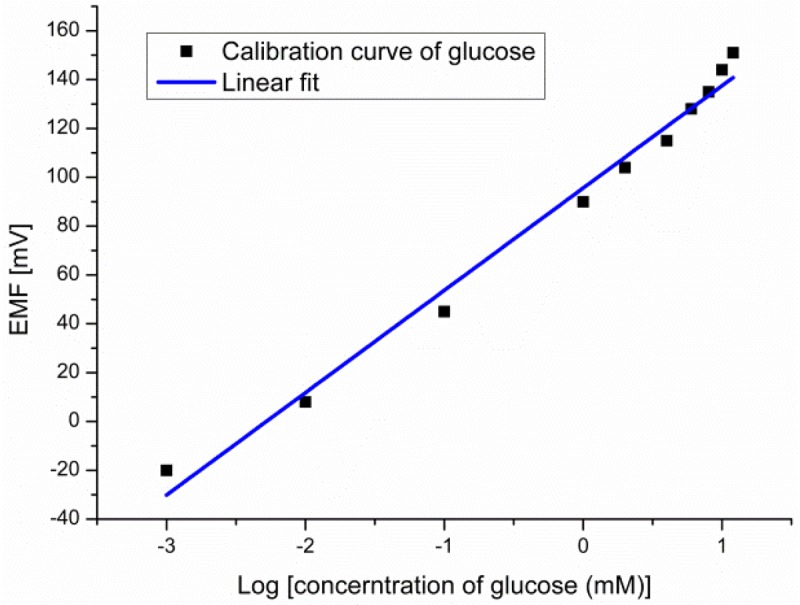
The calibration curve for glucose concentrations.

The EMF response of the glucose biosensor was measured between the working electrode and the reference electrode for different glucose concentrations. The experimental results showed large dynamic ranges over glucose concentrations going from 10^−3^ mM to 10 mM with a linear output response *vs.* logarithmic concentrations of glucose with a slope of 41.9 mV/decade as shown in Figure 6.

### 3.6. Inhibition Studies

**Figure 7.**
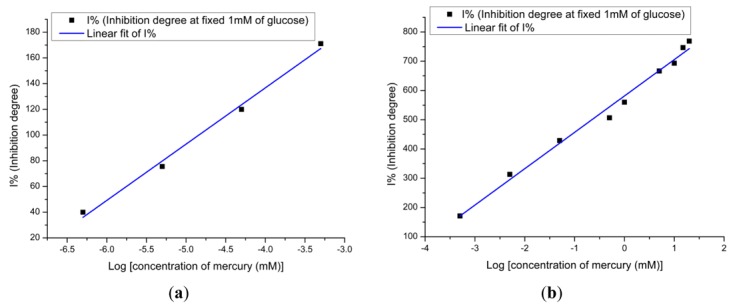

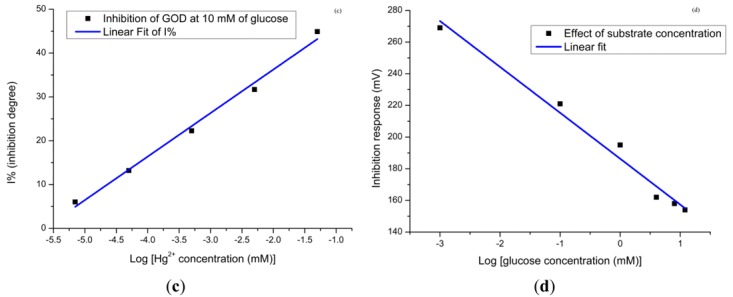
(**a**) A calibration curve for mercury ion inhibition at low glucose concentration and (**b**), (**c**) at high glucose concentration; (**d**) the effect of glucose concentration on degree of inhibition.

The enzyme–inhibitor reaction is often complex. The inhibition reactions between the enzyme and the toxic compound are reversible and irreversible inhibition. The mechanism of glucose oxidase inhibition by heavy metals is reversible and it makes the inhibitor bind at a site other than the active site of the enzyme and causes changes in the shape of the enzyme that lead to a change in the activity and a mixed inhibition cannot be overcome at high substrate concentration [34,35]. The degree of inhibition of the glucose oxidase is given by the following expression:

(2) $$I\%=100\left(\frac{I_{0}-I}{I_{0}}\right)$$

where I% represents the degree of inhibition, *I*_0_ represents the response to glucose in the absence of inhibitor and *I* represents the response to glucose in the presence of inhibitor [12,34,35]. In this work, after the stable response of sensor electrode in the standard PBS solution was obtained, an amount of low concentration glucose solution was added to give a final concentration of 1 mM glucose in the solution, and then its stable response was recorded (I_0_) after that solutions of higher concentrations of Hg^2+^ ions were added to inhibit the enzyme activity and the change in the output response (I) corresponding to the concentration of inhibitor in solution was recorded. The linear range *vs.* natural logarithm of Hg^2+^ ion concentration of the degree of inhibition of the GOD can be divided into two parts, as shown in Figure 7(a,b). In Figure 7(a), the linear range is from 0.5 × 10^−6^ mM to 0.5 × 10^−4^ mM and the correlation coefficient is 0.99. From Figure 7(b), the linear range is from 0.5 × 10^−4^ mM to 20 mM, also with a coefficient of 0.99. In order to study the effect of the substrate concentration on the inhibition of enzyme activity, a higher concentration 10 mM of glucose was also performed. The inhibition degree of the GOD at high concentration of the substrate is given by Figure 7(c).

From this graph we can see clearly that the linear range at the high substrate concentration is given from 10^−3^ mM to 6 mM. From these obtained results we can say that the lower concentration gives the lower limit of detection. Furthermore, the affected electrodes during the Hg^2+^ ions measurement were immediately used to measure the response of the glucose solution. Figure 7(d) shows the response in decreasing order while for the glucose concentration it is in increasing order. This is clear evidence that the higher substrate concentration provides a lower inhibition response. In order to perform multiple experiments, the activity of the enzyme at the surface of the electrodes has to be restored. To restore the enzyme activity, the used electrodes were washed with PBS after contact with the heavy metal. After this, for the electrodes used in inhibitor for 1 hour, the regeneration efficiency can reach up to 70% by dipping electrodes in 10 mM of PBS for 5 min. The biosensor activity could be restored more than 70% after dipping the used electrodes in PBS for more than 7 min, which demonstrates that Hg^2+^ ions are not strongly bound to the enzyme.

### 3.7. Selectivity

Selectivity is very important issue for the performance of a biosensor. In this study, we use the same approach as for the mercury for calculating the degree of inhibition. The influences of possible interfering metals such as Cu^2+^, Zn^2+^, Fe^2+^ and Co^2+^ were investigated in the presence of fixed amounts of 1 mM of glucose solution. The selective coefficient values for interference were calculated by the separation solution method (SSM) [36], using the following equation:

(3) $$\log K_{A,B}^{\text{pot}}=\frac{E_{B}-E_{A}}{S_{A}}+\left(1-\frac{S_{B}}{S_{A}}\right)\log(a_{A})$$

where A represents for target ion (Hg^2+^) and B represents for interference ions (Cu^2+^, Zn^2+^, Fe^2+^ or Co^2+^), E_A_ and E_B_ represent for EMF response for A and B at same 0.01 mM concentration, respectively, while S_A_ and S_B_ are the sensitivities of A and B, respectively. Finally, *a_A_* is the concentration of A. The calculated selectivity coefficient results are shown in Table 2. From Table 2 the calculated selectivity coefficient values of each interfering ion are fairly constant and the sensitivity to mercury ions is 1,000 times higher than for Cu^2+^ and Zn^2+^ and 100 times more than Fe^2+^ and Co^2+^. These values show that the interference effects are negligible.

### 3.8. Response Time, Reproducibility and Lifetime

**Figure 8.**
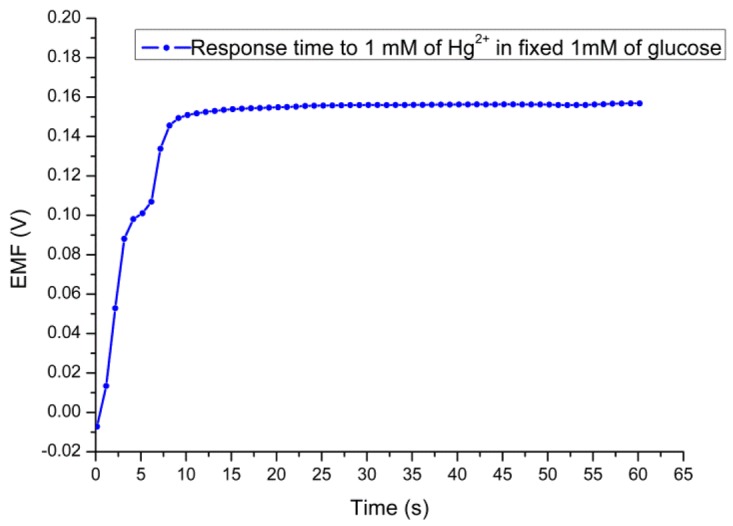
The response time of the proposed sensor.

**Figure 9.**
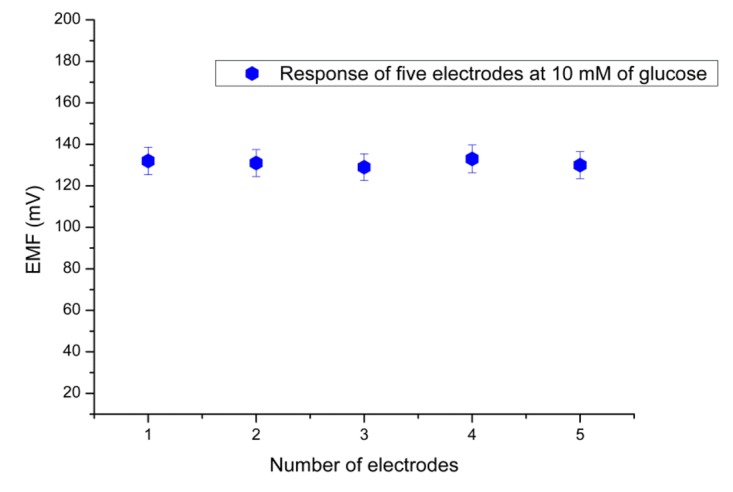
Reproducibility of the sensor electrodes.

Figure 8 shows a response time of 8 s for the proposed sensor in the presence of 1 mM of Hg^2+^ solution at a fixed concentration of 1 mM substrate concentration. The sensor to sensor reproducibility was evaluated by using five independent electrodes fabricated under the same conditions. The relative standard deviation of the fabricated sensor electrodes in standard glucose solutions was less than ±5% as shown in Figure 9. The stability of the presented biosensor has been investigated after the electrodes were stored for three weeks in dry conditions at 4 °C. It has been found that this biosensor has good storability and maintained 90% of its original sensitivity.

## 4. Conclusions

A potentiometric glucose biosensor made by immobilization of a glucose oxidase enzyme using physical adsorption in combination with chitosan membrane and GA on a ZnO-NRs electrode has been demonstrated. It has been observed that such an approach is good for measuring the inhibition effect of heavy metal ions on the enzyme activity, especially for measuring Hg^2+^ ion. The advantages of using ZnO-NRs, like e.g., suitability for detecting in small volumes with high sensitivity, low detection limit, fast response and ease of regeneration, are among the reasons of the developing such a sensor. The fabrication of this biosensor further has extra advantages like, e.g., being a low-cost approach, simple, requiring minimum hardware and possessing fast response times. Moreover, the presented sensor has demonstrated good sensitivity, reusability, reproducibility and good anti-interferent effects. Such a biosensor is also convenient for assembly into portable chips for chemical sensing. The proposed sensor electrode is also suitable for use by unskilled users and it can be applied to on-site measurement of mercury ions.

## Figures and Tables

**Table 1.** The performance of some glucose biosensors for mercury determination.

**Electrodes**	**Low Detection**	**Range (μM)**	**Response (s)**	**Anti-interference**	**Reference**
Carbon Paste Electrode	0.5 mg/L	2.0–32.5 mg/L	N/A	Cr^3+^ and Zn^2+^	[7]
Pt electrode	0.49 μg/L	0.49–783.21 μg/L and 783.21 μg/L–25.55 mg/L	60 s	Cr^3+^, Pb^2+^, Cu^2+^ and Cd^2+^	[8]
Thiolate self-assembled monolayer	0.2 ppb	1–100 ppb	N/A	Ascorbic acid, H_2_O_2_, Cu^2+^ and Zn^2+^	[9]
Pt/PPD/GOD	2.5 μM	5–180 μM	100 s	Cu^2+^, Cd^2+^, Co^2+^ and Ni^2+^	[10]

**Table 2.** The summary of calculated selective coefficient values.

**Interferences**	$\log K_{A,B}^{\text{pot}}$
Cu^2+^	−3.05226
Zn^2+^	−3.05009
Fe^2+^	−2.60417
Co^2+^	−2.86609
